# Short-Term Effects of Cerebellar tDCS on Standing Balance Performance in Patients with Chronic Stroke and Healthy Age-Matched Elderly

**DOI:** 10.1007/s12311-018-0939-0

**Published:** 2018-05-24

**Authors:** Sarah B. Zandvliet, Carel G. M. Meskers, Gert Kwakkel, Erwin E. H. van Wegen

**Affiliations:** 1 Department of Rehabilitation Medicine, VU University Medical Center, Amsterdam Neuroscience and Amsterdam Movement Sciences, Amsterdam, The Netherlands; 2 Department of Physical Therapy and Human Movement Sciences, Northwestern University, Chicago, IL USA; 3 Department of Neurorehabilitation, Amsterdam Rehabilitation Research Centre, Reade, Amsterdam, The Netherlands

**Keywords:** Transcranial direct current stimulation, Postural balance, Stroke, Cerebellum

## Abstract

Transcranial direct current stimulation (tDCS) may serve as an adjunct approach in stroke rehabilitation. The cerebellum could be a target during standing balance training due to its role in motor adaptation. We tested whether cerebellar tDCS can lead to short-term effects on standing balance performance in patients with chronic stroke. Fifteen patients with a chronic stroke were stimulated with anodal stimulation on the contra-lesional cerebellar hemisphere, ipsi-lesional cerebellar hemisphere, or sham stimulation, for 20 min with 1.5 mA in three sessions in randomized order. Ten healthy controls participated in two sessions with cerebellar stimulation ipsi-lateral to their dominant leg or sham stimulation. During stimulation, subjects performed a medio-lateral postural tracking task on a force platform. Standing balance performance was measured directly before and after each training session in several standing positions. Outcomes were center of pressure (CoP) amplitude and its standard deviation, and velocity and its standard deviation and range, subsequently combined into a CoP composite score (comp-score) as a qualitative outcome parameter. In the patient group, a decrease in comp-score in the tandem position was found after contra-lesional tDCS: *β* = − 0.25, CI = − 0.48 to − 0.03, *p* = 0.03. No significant differences in demographics and clinical characteristics were found between patients who responded (*N* = 10) and patients who did not respond (*N* = 5) to the stimulation. Contra-lesional cerebellar tDCS shows promise for improving standing balance performance. Exploration of optimal timing, dose, and the relation between qualitative parameters and clinical improvements are needed to establish whether tDCS can augment standing balance performance after stroke.

## Introduction

Recovery of standing balance after stroke is a key factor in regaining independence in activities of daily living (ADL) and preventing fall events [1]. A meta-analysis of interventions aimed to improve standing balance did not indicate superiority of a certain training method, suggesting the need for more effective interventions post-stroke [2].

Recently, non-invasive transcranial direct current stimulation (tDCS) has emerged as an innovative, promising approach in stroke rehabilitation [3]. tDCS may prime the brain before or during a therapeutic intervention, providing potential to augment the positive learning effects of task specific training, the idea being that such combined peripheral and central input enhances synaptic plasticity and skill relearning [4]. Recent literature, however, report inconsistent findings on improvement in motor performance when measured with clinical scales in patients with chronic stroke, suggesting that, if any effect exists at all, tDCS interventions may only induce subtle changes [5]. In addition, clinical outcomes such as gait speed and the Berg Balance Scale are not able to delineate between “true neurological repair” and behavioral compensation strategies [6]. Therefore, kinematic and kinetic measures are recommended in stroke recovery trials to demonstrate possible effects of tDCS in terms of quality of motor performance [6]. One may also argue that the subtle effects of tDCS, which are believed to enhance Hebbian and non-Hebbian learning processes by mechanisms of long-term potentiation (LTP) and long-term depression (LTD)-like plasticity [7], may induce most benefit in those brain areas that are responsible for learning-dependent motor control such as the cerebellum.

The cerebellum is known to be involved in error-based motor learning, also referred to as motor adaptation [8]. LTD-like plasticity of Purkinje cells is associated with learning, this Hebbian process that is mediated by simultaneous activation of parallel fibers and climbing fibers that give input to error signals in motor control to the cortex [9–11]. Balance performance can be seen as adaptation of posture [12], and the cerebellar hemispheres play a specific role in motor adaptation [13, 14]. A strong M1–cerebellar connection also results in more accurate movement endpoints, emphasizing the crucial role of the cerebellum in motor adaptation [8, 12, 15]. The more medial flocculonodular lobe of the cerebellum is directly linked to postural balance [16], but can likely not be targeted with tDCS due to its anterior location, while the hemispheres can be targeted with tDCS [17].

Cathodal stimulation of a cerebellar hemisphere leads to a decrease in cerebellar brain inhibition, likely via an enhancement of LTD of Purkinje cells [18, 19]. In healthy subjects, cathodal stimulation of the cerebellum has been found to lead to improvement of balance performance in the study of Inukai et al. [20], while Foerster et al. [21] found an impairing effect. Anodal stimulation has been found to lead to significant improvements in motor adaptation and balance performance in several studies in healthy subjects, while others found no added value of stimulation [14, 20–24]. The potential benefits of cerebellar tDCS in stroke patients with balance impairments have to date not been investigated.

It has been suggested that anodal cerebellar tDCS (cb_tDCS) enlarges the population of activated Purkinje cells, leading to a larger involvement of the cerebellum in the executed motor task [23]. More recent animal studies also showed that synaptic-based forms of learning cannot take place in the absence of LTP of Purkinje cells, suggesting a complex interplay of LTD and LTP in the cerebellum [7, 25–27]. Bearing in mind the function of the cerebellum, as controller of temporal and spatial accuracy, it can be argued that both Hebbian and anti-Hebbian processes play a role in the cerebellum and optimization of these processes can lead to an enhancement of motor adaptation. It is plausible that a positive effect of stimulation can therefore only be found if there is a need for improvement of balance performance, which is unlikely in healthy young adults; however, there is a substantial clinical problem in patients with a stroke [1, 2, 24].

In this proof-of-concept study, we investigated for the first time the short-term effects of anodal cb_tDCS applied on both the ipsi-lesional as well as the contra-lesional cerebellar hemisphere as compared to sham stimulation during a postural training task in patients with chronic stroke and healthy age-matched controls. Since clinical tests are not sensitive enough to record the qualitative aspects of balance performance and with that, unable to record the subtle changes that can be expected from a single session of tDCS, the effects are measured with kinetic parameters. We hypothesized that both anodal stimulation conditions normalize standing balance performance measured with center of pressure (CoP)-derived parameters in patients with stroke. Normalization of standing balance performance was defined as a decrease in CoP parameters in patients with a stroke. The largest effect on standing balance performance was expected in the most difficult task, a (semi-)tandem stance, due to the expected room for improvement via motor adaptation, especially in the group of patients with a stroke.

## Methods

Measurements took place at the rehabilitation department of the VU University Medical Center. All procedures performed in this study involving human participants were in accordance with the ethical standards of the institutional research committee and with the 1964 Helsinki declaration and its later amendments and approved under reference: NL52021.029.15. All subjects gave their written informed consent.

### Subjects

Fifteen patients with chronic stroke (> 6 months post-stroke) and ten age– and gender-matched healthy controls were enrolled in this study. Patients had to meet the following criteria to participate:

1. A first ever ischemic or hemorrhagic lesion excluding lesions of the cerebellum as verified by CT or MRI scan,
2. Decreased standing balance performance as determined by a score of < 56 points on the Berg Balance Scale (BBS).

In addition, all patients and healthy controls had to meet the following additional criteria:

1. Age ≥ 18,
2. Normal or corrected to normal vision with an optical aid,
3. Able and sufficiently motivated to perform the required tests and interventions,
4. No metallic implants near to the side of stimulation,
5. No orthopedic limitations that interfere with the study,
6. No cranial bone defects,
7. No history of epileptic seizures,
8. No psychiatric disorder,
9. No signs of depression (Hospital Anxiety and Depression Scale, HADS, subscore *D* < 7) [28],
10. Sufficient cognitive function (Mini Mental State Examination, MMSE ≥ 19),
11. No sensory impairments (prior to the ischemic lesion, in case of patients),
12. No diagnosed diseases of the vestibular system,
13. Absence of additional therapy focusing on standing balance improvement during the time period in which the measurements took place,
14. No history of disease, condition, event, or use of medication that interfered with the study.

### Protocol

**Fig. 1:**
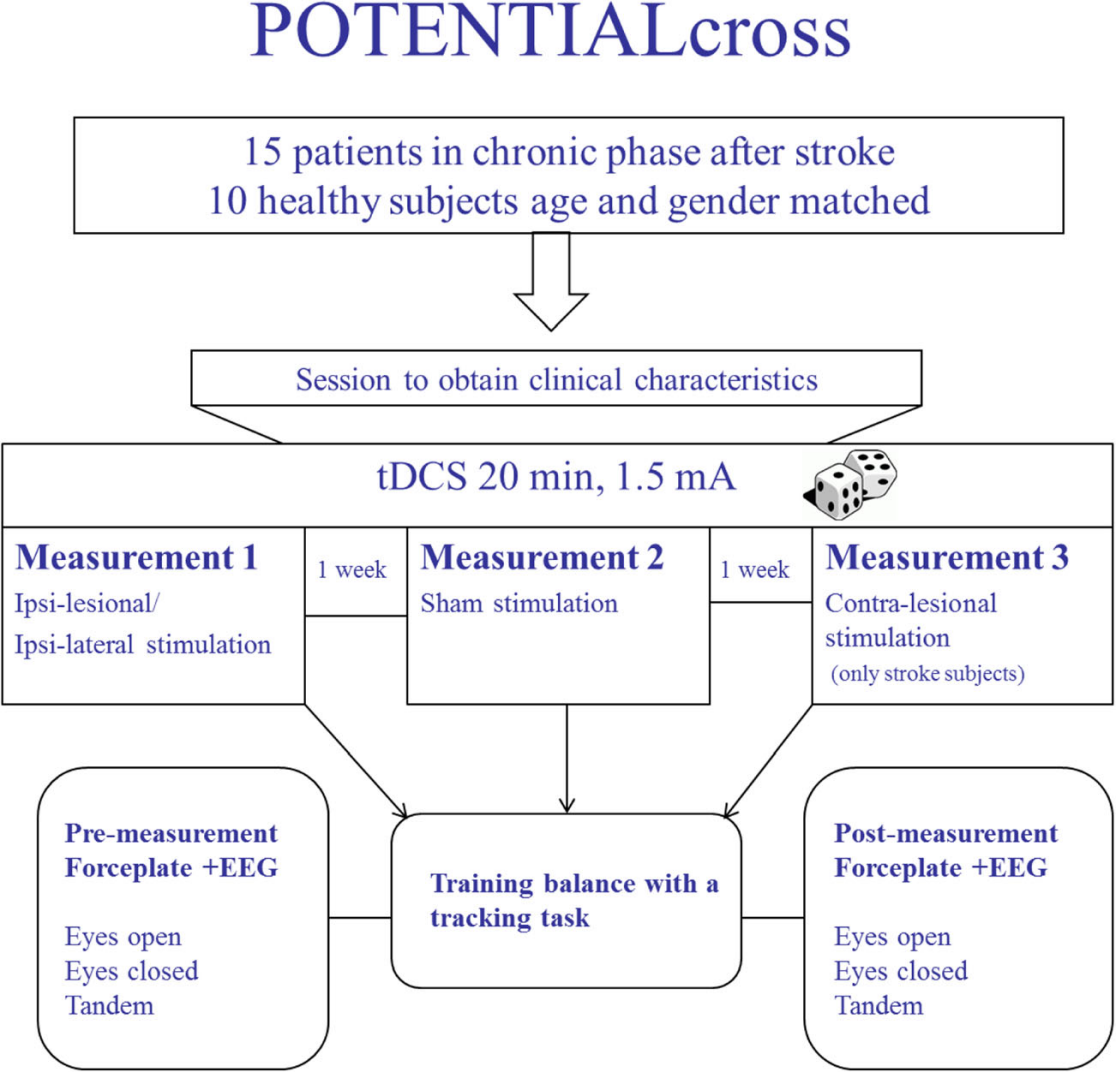
Overview of the experimental protocol. Patients were stimulated in one session at the contra-lesional side, and in one session at the ipsi-lesional side. Sham stimulation was applied to the contra-lesional side. Healthy controls had two sessions both ipsi-lateral to their dominant leg with either real or sham stimulation

All subjects first participated in an intake and clinical assessment session, after which they returned for respectively two (healthy controls) or three (patients) sessions, in which they had to perform a medio-lateral postural tracking task while being stimulated with anodal cb_tDCS. Directly before and after this tracking task with cb_tDCS, standing balance performance was assessed during several quiet standing tests. These sessions were minimally one and maximally two weeks apart. For a schematic overview of the protocol, see Fig. 1.

### Tracking Task

**Fig. 2:**
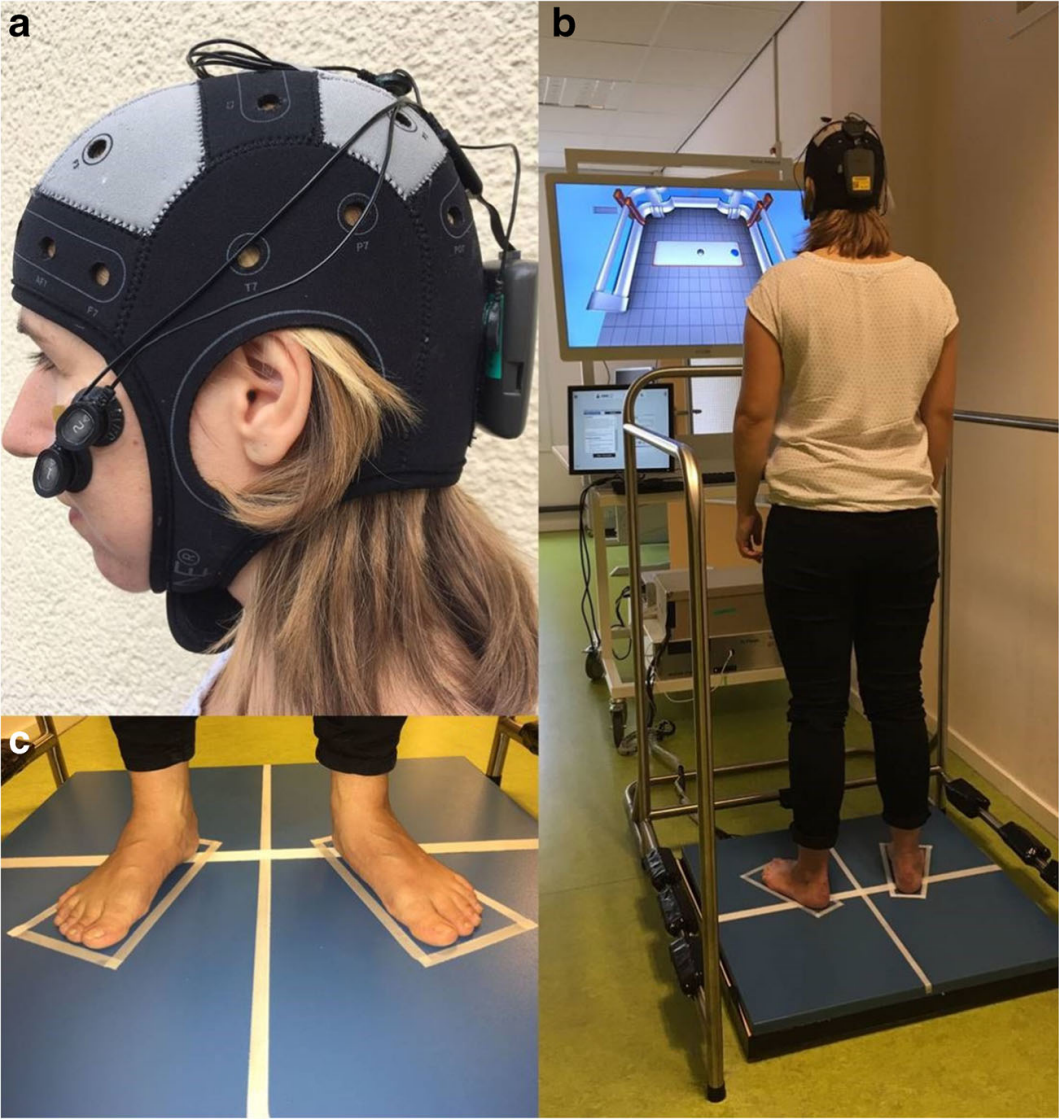
Overview of experimental setup. **a** Cerebellar transcranial direct current stimulation was delivered by a portable stimulator with 1.5 mA current for 20 min in all sessions. **b** Subject performing the tracking task on a Balance Workstation (Motek). The blue dot represented a moving target, which the subject was asked to follow as precisely as possible with the gray tracking dot, representing their center of pressure measured with the force plate. **c** Subjects stood with their feet at hip width in 9° degrees exorotation for five times 1 min per standing position. The position was marked for repositioning after rest

Two dots were presented on a video screen located at eye height for the tracking task (Fig. 2b). One dot represented a moving target, which the subject was asked to follow as precisely as possible with the second tracking dot, representing their CoP measured with a force plate. The CoP signal was low pass filtered with a second-order Butterworth filter at 10 Hz (D-flow, Balance Workstation, Motek, Amsterdam, The Netherlands). Four repetitions of the tracking task of 3 min each were performed by each subject. In two of those repetitions, the target moved in a predictable manner with an increasing velocity in eight blocks of 20 s from 0.16 to 1.28 cm/s. During the two other repetitions, the target moved in a pseudo-random manner with 16 blocks of 10 s with a velocity between 0.16 and 1.28 cm/s. Those repetitions were therefore considered to be unpredictable in terms of velocity. Subjects received feedback on their performance after each repetition. Performance was defined as the percentage of the time of accurate overlap of the target and tracking dot during the eight different velocities. Subsequently, subjects could rest as long as needed between repetitions.

### Performance on the Tracking Task

To evaluate a possible learning effect of the tracking task, performances of the last two repetitions were averaged over all frequencies. This included one repetition with a predictable and one with an unpredictable moving target.

### tDCS

During the tracking task, cb_tDCS was delivered by a battery-driven portable stimulator (Starstim ®; Neuroelectrics, Barcelona, Spain) through 3.14-cm^2^ electrodes filled with conducting gel, with a direct 1.5 mA current. The anodal electrode was placed 3 cm lateral of the inion. Two cathodal electrodes were placed on the ipsi-lateral buccinators muscles (Fig. 2a). Anodal stimulation was applied for 20 min with 1.5 mA in all sessions. During the first 3 min of stimulation, subjects were sitting on a chair to get acquainted with the stimulation while the tracking task was explained. For patients, three experimental sessions, with minimally one week and maximally two weeks apart, were performed: (1) anodal cb_tDCS on contra-lesional side, (2) anodal cb_tDCS on the ipsi-lesional side, and (3) sham cb_tDCS on the contra-lesional side. In sham tDCS, the current was automatically switched off after 30 s and switched on again for the last 30 s of the 20-min stimulation period. Healthy controls only had two sessions, both ipsi-lateral to their dominant leg, with either anodal or sham stimulation. The order of the sessions was randomized. The stimulation was always finished before subjects finished their fourth repetition of the tracking task. The dominant leg was determined by the preferred leg used and better performance on the lateralized items of the BBS (i.e., looking over the shoulder, turning, tandem stance, and one-leg stance).

### Standing Balance Performance Assessment

Before and after the stimulation, standing balance performance was assessed during several quiet standing positions. Ground reaction forces were measured during these positions with a sample frequency of 1000 Hz using an 80 × 80 cm force plate (Motek, Amsterdam, The Netherlands). The analog signals were converted with a 16-bit analog-to-digital converter with a 10-V range (National Instruments, Austin, USA).

Subjects were asked to stand quietly in three sequential standing positions: (1) with eyes open, (2) eyes closed, and (3) in the most challenging, subject-specific (semi-) tandem stance, on a force plate during five repetitions of 1 min. Subjects rested for a minimum of 30 s in between standing tests. The rest period was extended upon the subjects’ request to avoid fatigue. In standing positions 1 and 2, subjects stood barefoot with their arms relaxed, alongside the trunk if possible, with their feet at hip width in 9° exorotation (Fig. 2c). During the tandem stance, subjects were asked to stand in the most difficult position which they could hold for 1 min as determined in the first clinical assessment session. The foot positions of the first session were marked and measured to keep foot placement the same in the consecutive sessions.

At four time points during a session, subjects were asked to indicate their level of headache, nausea, fatigue, and depressed mood on a visual analog scale (VAS) from 0 to 100 mm, in order to track commonly reported side effects of tDCS [29].

All subjects were told at the end of all measurements that during one of the sessions, a placebo stimulation was given and asked if they could indicate which session it was.

### Clinical Assessments

The clinical measures were performed by the researcher according to recommended guidelines [2] before the sessions with tDCS. The following assessments were performed: Berg Balance scale (BBS) [30], functional reach task (FRT) [31], timed up and go (TUG) [32], fall efficacy scale (FES) [33], fall history, and Erasmus modification of the Nottingham Sensory Assessment of the Lower Extremity (EmNSA-LE) [34]. For healthy controls, the dominant leg was determined by the preferred leg used and better performance on the lateralized items of the BBS (i.e., looking over the shoulder, turning, tandem stance, and one-leg stance). The most difficult position for subjects to hold for more than 30 s was determined with an ordinal scale ranging from (1) full tandem stance with non-paretic leg/dominant in front, (2) non-paretic/dominant leg a step ahead, (3) non-paretic/dominant leg half a step ahead, (5) full tandem stance with paretic leg/non-dominant in front, (6) paretic/non-dominant leg a step ahead, (7) paretic/non-dominant leg half a step ahead, or (8) feet placed together.

Stroke patients were also assessed with the Fugl–Meyer Motor Assessment of the Lower Extremity (FMA-LE) [35], Motricity Index (MI) [36], Nottingham Extended Activities of Daily Living index (NEADL) [37], and O-Letter Cancelation test (LCT) [38].

### Data Analysis and Pre-Processing

Recorded data were processed using MATLAB 2012a (the MathWorks, Inc., Natick, MA, USA). Statistical analysis was performed using IBM SPSS statistics version 22 (IBM Corporation, Armonk, NY, USA).

### Outcome Parameters

Force plate data were low pass filtered with a second-order Butterworth filter with a cutoff frequency of 10 Hz, after which the CoP in both the anterior–posterior (AP) and the medio-lateral (ML) direction was calculated. The middle 50 s of each trial were used for further analysis. The signals were linear detrended with a period of 20 s. The following parameters were computed from the sum of the vectors of the CoP in the AP and ML direction: the mean amplitude of the CoP (ACoP) calculated as the root mean squared distance from the mean CoP, its standard deviation represents the amplitude’s variability (varCoP), the velocity of the CoP (VCoP) calculated by the sum of the distance between sequential points divided by its length, its standard deviation represents the velocity’s variability (varVCoP), and the range determined as the maximal difference between any two points of the time series. A CoP composite score of the abovementioned parameters was calculated as a comprehensive outcome parameter representing qualitative aspects of standing balance performance [39]. Each parameter, calculated for ML and AP direction separately, was transformed to a *z*-score calculated over all parameters, separately for the stroke patients and the healthy controls. This transformation leads to a mean of 0 with a standard deviation of 1, making it possible to average the ten transformed parameters per standing position into a CoP composite-score (comp-score).

### Statistical Analysis

Normality was checked by visual inspection of the probability distribution (q–q plot) and the box plot. A Shapiro–Wilks test was performed on the data or the residual when appropriate. When the assumptions of normality were not met, a natural log transformation was applied after which normality was checked again. In case this transformation was not sufficient, or in case of ordinal or nominal data, the non-parametric equivalent of the below mentioned tests was used. The significance level *α* was set two-tailed at 0.05.

CoP baseline differences between healthy controls and patients were analyzed with an independent *t* test with a Bonferroni correction for multiple testing per standing position. To correct for differences in sample size, Hedges’ *g* (*g*) was used to calculate effect sizes, when significant differences were found.

A generalized estimating equation (GEE) model, with a correction for baseline CoP comp-score, was used to establish an association between the stimulation conditions on CoP comp-scores for patients and healthy control separately. The model was also tested for confounding of randomization order. If the *β* values for the stimulation conditions changed with more than 10%, this was considered an improvement of the model. In case of a significant association between post-measurement CoP comp-score and cb_tDCS, the parameters from which the CoP comp-score was constructed were evaluated separately.

### Performance on the Tracking Task

A GEE model was used to evaluate the improvement of performance on the tracking task over time. Stimulation condition was added as a possible confounder to examine if performance was influenced by cb_tDCS.

### Responders and Non-Responders

To investigate the differences in response to cb_tDCS between subjects (interindividual response), a distinction was made between patients who showed a response on the CoP comp-score and patients who did not. A subject was defined as a “responder” when a change in CoP comp-score (post–pre) for the tandem stance was more than one standard deviation larger in the contra-lesional stimulation or the ipsi-lesional condition compared to the sham condition. Differences in baseline CoP comp-score, fatigue, and clinical and demographic characteristics between responders and non-responders were analyzed with independent *t* tests.

## Results

### Procedures

All subjects completed the experimental protocol. One patient was not able to perform five repetitions of each static assessment of standing balance performance due to fatigue. Three repetitions of each assessment were performed instead.

Two patients were able to perform a semi-tandem stance for 60 s with the non-paretic leg ahead of the paretic leg, ten patients were able to perform a semi-tandem stance with the non-paretic leg a small step in front of the paretic leg, and three patients were able to perform a semi-tandem stance with the paretic leg a small step in front of the non-paretic leg. Nine healthy controls were able to perform a tandem stance with the dominant leg ahead, and one healthy control performed a semi-tandem stance with the dominant leg ahead.

### Successfulness of Blinding Procedures

When asked to indicate the sham condition at the end of the protocol, four out of ten healthy controls answered correctly, five incorrectly, and one could not make a choice. From the patients, seven answered correctly, six incorrectly, and two patients could not make a choice.

### Possible Side Effects, Fatigue

No subjects reported headaches or nausea during any of the sessions. Three healthy controls reported to be somewhat fatigued, but no differences in VAS on fatigue between sham: median (md) = 0, interquartile ranges (iqr) = 0–0.15 and cb_tDCS: md = 0, iqr = 0–0, were found, *z* = − 0.54, *p* = 0.60. Eleven patients reported a higher VAS on fatigue after stimulation, but no differences were found between the sham: md = 1.1, iqr = 0–3; contra-lesional: md = 1, iqr = 0–2.6; and ipsi-lesional: md = 1.3, iqr = 0–2.1, *χ*^2^ = 0.98, *p* = 0.61 conditions. Only one patient reported 7 out of 100 points on the VAS for depressed mood at the start of one of the sessions; no increase was reported after stimulation.

### Baseline Differences Between Patients and Healthy Controls

Characteristics of both groups are displayed in Table 1. Healthy subjects and patients did not differ significantly in age, weight, and height. Patients had a lower score on the BBS md = 50, iqr = 48–53, as well as on the EmNSA-LE: md = 38, iqr = 34–39 and were slower on the TUG: mean = 14.3 s, sd = 7.9 s, compared to healthy controls (BBS—md = 56, iqr = 56–56, *p* < 0.01; EmNSA-LE—md = 39, iqr = 39–40, *p* = 0.02; TUG—mean = 6.1, sd = 0.99, *p* < 0.01).

**Table 1:** Baseline characteristics and clinical assessments

Subjects’ characteristics	Stroke subjects *N* = 15	Healthy subjects *N* = 10	*p* value
Gender, male/female	12/3	6/4	0.29
Age in years (mean, sd)	57.1 (10.0)	57.9 (7.1)	0.82
Weight in kilograms (mean, sd)	86.1 (21.1)	78.2 (9.24)	0.41
Height in meters (mean, sd)	1.78 (0.10)	1.78 (0.73)	0.84
Time since stroke in months (mean, sd)	107.8 (143.6)	–	–
Affected hemisphere, right/left	9/6	–	–
Cortical/sub-cortical stroke	13/2		
Bamford classification, LACI/PACI/TACI/unknown	7/4/2/2	–	–
Type of stroke, ischemic/hemorrhagic	11/4	–	–
CIRS, range 0–52 (median, iqr)	5 (4–6)	–	–
HADS, range 0–42 (median, iqr)	4 (3–10)	3.5 (0.75–5.25)	0.30
BBS, range 0–56 (median, iqr)	50 (48–53)	56 (56–56)	< 0.01
TUG in seconds (mean, sd)	14.3 (7.9)	6.1 (0.99)	< 0.01
EmNSA-LE, range 0–40 (median, iqr)	38 (34–39)	39 (39–40)	0.02
Falls past 6 months (median, iqr)	1 (0–2)	0.5 (0–1)	0.39
FES, range 7–28(median, iqr)	10 (8–12)	–	–
FM-LE, range 0–34 (median, iqr)	25 (22–30)	–	–
MI-LE, range 0–100 (median, iqr)	69 (58–83)	–	–
MI-UE, range 0–100 (median, iqr)	84 (76–93.75)	–	–
Spatial neglect, yes/no	5/10	–	–

Overview of baseline characteristics and clinical assessments measured in the first session for 15 the patients and 10 healthy subjects. Mean per group are given as well as the standard deviation (sd) or the median and inter quartile ranges (iqr) in case of ordinal scales and the frequencies in case of nominal data. Deviation in cortical and sub-cortical lesions are made base on the main classification made by a clinician directly after stroke

*TACI* total anterior circulation infarct, *PACI* partial anterior circulation infarct, *LACI* lacunar anterior circulation infarct, *CIRS* Cumulative Illness Rating Scale, *HADS* Hospital Anxiety and Depression Scale, *BBS* Berg Balance Scale, *TUG* Timed Up and Go, *EmNSA-LE* Erasmus modification of the Nottingham Sensory Assessment Lower Extremity, *FES* Fall Efficacy Scale, *FM-LE* Fugl–Meyer assessment lower extremity, *MI-LE* Motricity Index of the Lower Extremity, *MI-UE* Motricity Index of the Upper Extremity, *N* number per group

**Fig. 3:**
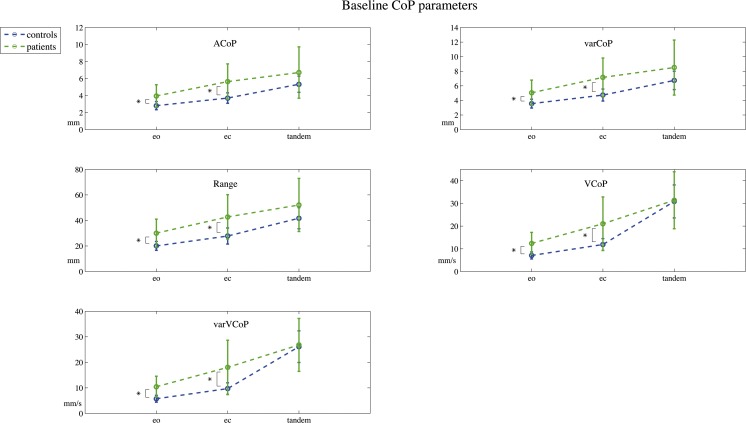
Baseline CoP parameters. Baseline center of pressure (CoP) parameters measured during the pre-stimulation eyes open (eo), eyes closed (ec), and a subject-specific tandem stance (tandem) position. The mean amplitude of the CoP (ACoP) and its amplitude’s variability (varCoP), and the velocity of the CoP (VCoP) and the velocity’s variability (varVCoP) are displayed. Error bars indicate the standard deviation of the mean per group. *Indicates a significant difference (probability value < 0.05) between patients and healthy controls

The baseline CoP parameters are displayed in Fig. 3. Patients showed significant larger excursion on all baseline CoP parameters for the eyes open; ACoP: mean healthy = 2.90 mm, sd = 0.47, mean patients = 4.17 mm, sd = 1.53, CI of the mean difference = − 2.16 to − 0.38, *t*(17.6) = − 3.01, *g* = 1.00; varCoP: healthy = 3.67 mm, sd = 0.58, patients = 5.35 mm, sd = 1.99, CI = − 2.83 to − 0.53, *t*(17.3) = − 3.07, *g* = 1.02; range: healthy = 20.68 mm, sd = 0.22, patients = 32.21 mm, sd = 0.42, CI = − 18.88 to − 4.17, *t*(16.4) = − 3.31, *g* = 31.37; VCoP: md healthy = 7.10 mm/s, iqr = 5.88–7.81, md patients = 10.78 mm/s, iqr = 8.80–15.48, CI = 0.43–0.78, ratio due to log transformation, *t* (23) = 0.02, *g* = 1.71; varVCoP: md healthy = 5.79 mm/s, iqr = 5.79–6.62, patients = 8.81 mm/s, iqr = 7.76–13.87, CI = 0.40 to 0.75, ratio due to log transformation, t(23)=0.02, *g* = 1.57, all *p* < 0.05.

This was also the case for the eyes closed position when compared to healthy age-matched controls; ACoP: mean healthy = 3.66 mm, sd = 0.65, mean patients = 5.71 mm, sd = 2.02, CI = − 3.23 to − 0.88, t(18) = − 3.67, g = 1.22; varCoP: healthy = 4.66 mm, sd = 0.83, patients = 7.21 mm, sd = 2.57, CI = − 4.06 to − 1.07, *t* (18)= − 3.60, *g* = 1.20, range: healthy = 26.49 mm, sd = 5.23, patients = 42.16 mm, sd = 15.20, CI = − 24.59 to − 6.74, *t*(18.5) = − 3.68, *g* = 1.23; VCoP: healthy = 11.62 mm/s, sd = 2.28, patients = 20.44 mm/s, sd = 8.91, CI = − 13.91 to − 3.73, *t*(16.6) = −3.66, *g* = 1.20; varVCoP: healthy = 9.42 mm/s, sd = 1.94, patients = 17.26 mm/s, sd = 7.56, CI = − 12.16 to − 3.51, *t*(16.6) = − 3.83, *g* = 1.26, all *p* < 0.05. For the (semi-)tandem stance position, no significant differences were found between patients and healthy controls; ACoP: md healthy = 5.17 mm, iqr = 4.65–6.34, md patients = 5.86 mm, iqr = 4.65–7.89, ratio due to log transformation, CI = 0.63 to 1.15, *t* (23) = 0.33; varCoP: md healthy = 6.55 mm, iqr = 5.80–8.45, md patients = 7.31 mm, iqr = 5.82–10.09, ratio due to log transformation, CI = 0.63 to 1.15, *t*[23]=0.33; range: md healthy = 40.29 mm, iqr = 36.65–55.94, md patients = 47.72 mm, iqr = 33.14–64.73, ratio due to log transformation, CI = 0.64 to 1.17, *t*(23) = 0.37; md VCoP: healthy 30.25 = mm/s, iqr = 26.22–32.48, md patients = 26.33 mm/s, iqr = 23.90–36.83, U = 61, *z* = − 0.78; varVCoP: md healthy 26.24 = mm/s, iqr = 21.40–28.96, md patients = 23.84 mm/s iqr = 19.47–31.16, *U* = 71, *z* = − 0.22, all *p* > 0.5.

### Effect of Stimulation on CoP Parameters

The tested model revealed no significant changes in CoP comp-score associated with cb_tDCS in the eyes open: *β* = 0.02, CI = − 0.09 to 0.12, *p* = 0.73; eyes closed: *β* = 0.08, CI = − 0.01 to 0.16, *p* = 0.07; and tandem: *β* = − 0.08, CI = − 0.41–0.25, *p* = 0.64 for the healthy controls, see Table 2. Adding the stimulation order to the model did not change *β* values with more than 10%.

**Fig. 4:**
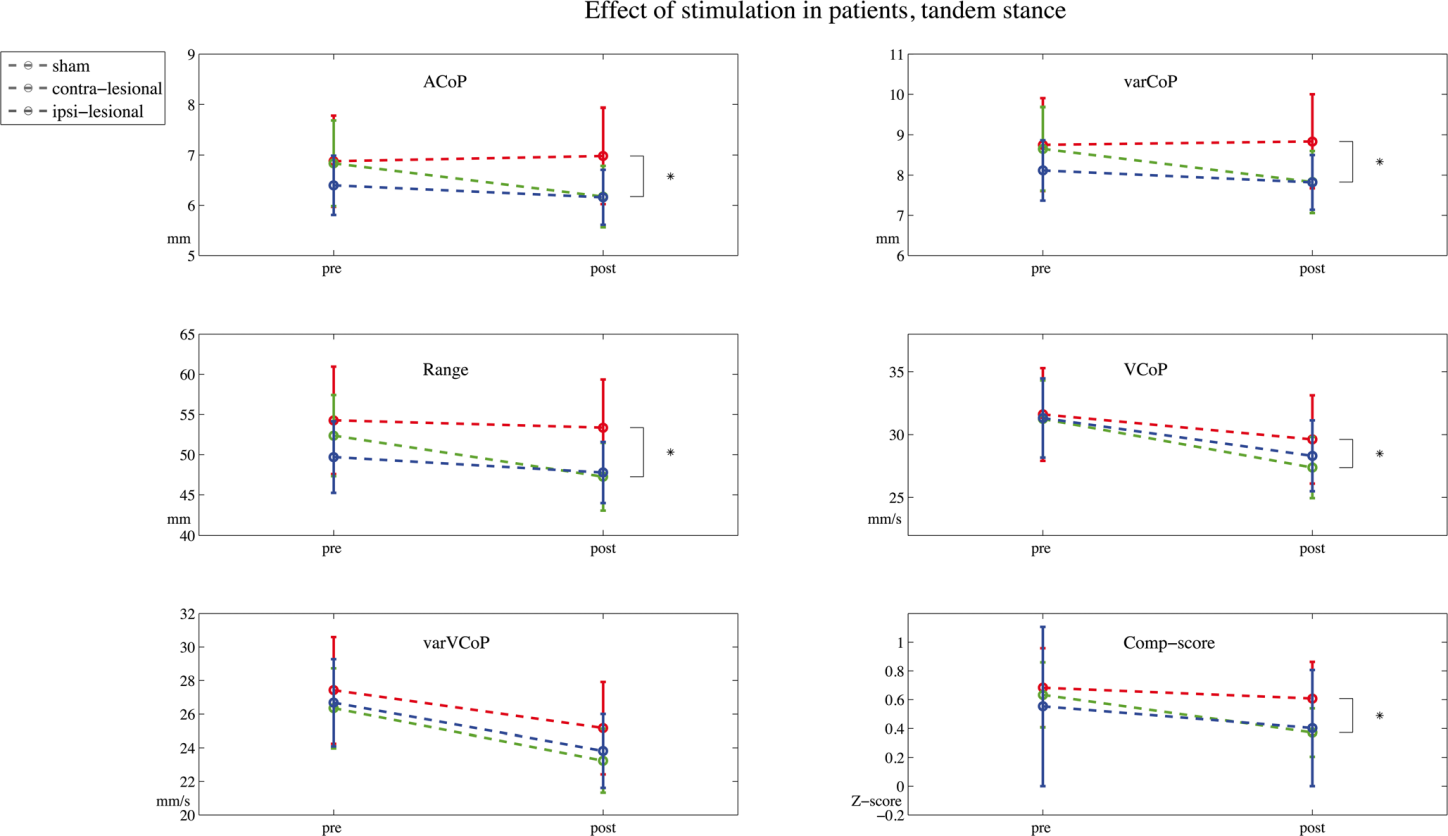
Effect of stimulation on tandem stance performance in stroke. Center of pressure (CoP) parameters measured during the pre-stimulation and post-stimulation in the subject-specific tandem stance (tandem) positions. The mean amplitude of the CoP (ACoP) and its amplitude’s variability (varCoP), and the velocity of the CoP (VCoP) and the velocity’s variability (varVCoP) and de composite-score are displayed. *Indicates a significant difference with a probability value of < 0.05, in the generalized estimating equation model with a correction for baseline and randomization order between contra-lesional cerebellar transcranial direct current stimulation (cb_tDCS) and the sham condition. Error bars indicate the standard error of the mean

In the patient group, a significant association between contra-lesional stimulation and a decrease in CoP comp-score in the tandem position was found: *β* = − 0.25, CI = − 0.48 to − 0.03, *p* = 0.03. Post hoc analysis showed a significant decrease in ACoP: *β* = − 0.86, CI = − 1.58 to − 0.15, *p* = 0.02; varCoP: *β* = − 1.10, CI = − 1.93 to − 0.26, *p* = 0.01; range: ratio due to log transformation, *β* = 0.94, CI = 0.90–0.98, *p* = 0.01; and VCoP: ratio due to log transformation, *β* = 0.97, CI = 0.94 to 0.99, *p* = 0.02 but not in varVCoP: ratio due to log transformation, *β* = 0.97, CI = − 0.93 to 1.01, *p* = 0.11, see Fig. 4. The GEE-model constructed for the eyes open position revealed a significant association between ipsi-lesional stimulation and a lower CoP comp-score: *β* = − 0.09, CI = − 0.18 to − 0.01, *p* = 0.03; after correcting for randomization order, the association was no longer significant and changed to *β* = 0.00, CI = − 0.09 to 0.90, *p* = 0.94.

**Fig. 5:**
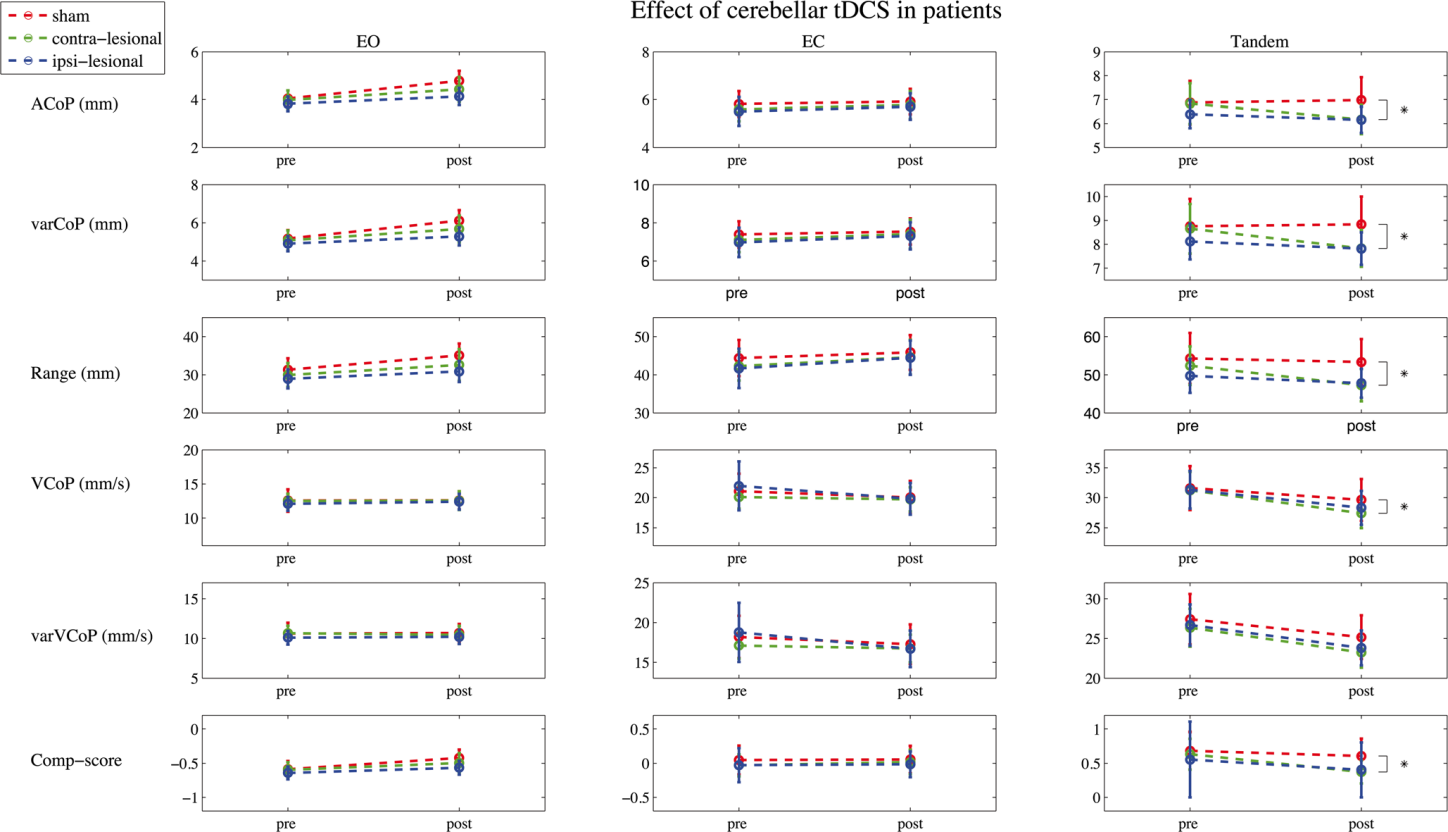
CoP parameters measured pre- and post-stimulation in stroke patients. Centre of pressure (CoP) parameters measured during the pre-stimulation (pre) and post-stimulation (post) in the eyes open (eo), eyes closed (ec), and a subject-specific tandem stance (tandem) position. The mean amplitude of the CoP (ACoP) and its amplitude’s variability (varCoP), and the velocity of the CoP (VCoP) and the velocity’s variability (varVCoP) and the composite-score (Comp-score) are displayed. *Indicates a significant difference (probability value < 0.05) in the generalized estimating equation model with a correction for baseline and randomization order between contra-lesional cerebellar transcranial direct current stimulation (cb_tDCS) and sham. Error bars indicate the standard error of the mean. Note that the *y*-axis between conditions differs for visual inspection purposes

No changes in CoP comp-score in the eyes closed position associated with cb_tDCS were found for patients, contra-lesional stimulation: *β* = 0.02, CI = − 0.11–0.16, *p* = 0.73 and ipsi-lesional stimulation: *β* = − 0.01, CI = − 0.19–0.16, *p* = 0.89. Adding the stimulation order to the model changed *β* values with more than 10% to contra-lesional stimulation: *β* = 0.06, CI = − 0.10–0.22, *p* = 0.44 and ipsi-lesional stimulation: *β* = 0.09, CI = − 0.08–0.26, *p* = 0.30 (Fig. 5). See Table 2 for an overview of the corresponding *β* values and confidence intervals.

### Performance on the Tracking Task

The healthy controls showed a significantly higher tracking task performance during the second measurement, m = 99.33, sd = 0.57, *β* = 0.80, CI = 0.10 to 1.50, *p* = 0.03, as compared to the first measurement, m = 98.53, sd = 1.31. No effect of stimulation was found *β* = 0, CI = − 0.70 to 0.70, *p* = 1.0.

The patient group showed a significantly higher tracking task performance during the second measurement; m = 92.90, sd = 7.5, with a ratio of *β* = 1.62, CI = 1.23 to 2.14, *p* < 0.01 and on the third measurement, m = 94.60, sd = 6.11, with a ratio of *β* = 2.06, CI = 1.51 to 2.80, *p* < 0.01 as compared to the first measurement, m = 88.60, sd = 9.29.

No effect of contra-lesional stimulation was found with a ratio due to log transformation of *β* = 1.15, CI = 0.89 to 1.50, *p* = 0.29, nor an effect of ipsi-lesional stimulation, *β* = 1.16, CI = 0.91–1.49, *p* = 0.23.

### Responders and Non-responders

Ten patients had a reduction in CoP comp-score in the tandem stance in response to contra-lesional stimulation (responders). Five patients did not show a change when compared to sham stimulation (non-responders). The group responders did not differ on any of the baseline CoP comp-scores, fatigue level, or clinical characteristics from the non-responders (see Table 3). Out of the five non-responders, two patients could be identified as responders on the ipsi-lesional stimulation, while five patients responded to both stimulation types.

**Table 3:** Differences in characteristics and clinical assessments between responders and non-responders

Subject characteristics	Responders *N* = 10	Non-responders *N* = 5	*p* value
Age in years (mean, sd)	54.4 (± 8.88)	62.4 (± 10.94)	0.15
Time since stroke in months (mean, sd)	76.9 (± 86.2)	169.8 (± 219.8)	0.25
Affected hemisphere, right/left	5/5	4/1	0.18
Bamford classification, LACI/PACI/TACI/unknown	4/3/1/2	3/1/1/0	0.23
Type of stroke, ischemic/hemorrhagic	9/1	2/3	0.07
CIRS, range 0–52 (median, iqr)	5 (3.75–6.25)	5 (3.5–7)	1.00
HADS, range 0–42 (median, iqr)	4 (3–10.25)	5 (1.5–8)	0.85
BBS, range 0–56 (median, iqr)	50 (48.75–52.25)	52 (43–53.5)	0.76
TUG in seconds (mean, sd)	11 (8.75–17)	15 (8–20.5)	0.67
EmNSA-LE, range 0–40 (median, iqr)	38 (35.5–39.25)	37 (30.5–39)	0.46
FES, range 7–28 (median, iqr)	10 (7.75–12.5)	11 (7.5–14)	0.95
Falls past 6 months (median, iqr)	1 (0–1.5)	2 (0–2)	0.70
MI-LE, range 0–100 (median, iqr)	26 (22.75–30.25)	24 (13–28)	0.30
MI-UE, range 0–100 (median, iqr)	70.5 (62.5–85)	59 (45.5–72)	0.16
Spatial neglect, yes/no	3/7	2/3	0.20
Fatigue VAS, range 0–100 (median, iqr)	2.2 (± 3.2)	1.6 (± 1.2)	0.70
CoP pre_comp_EO (mean *z*-score, sd)	− 0.43 (± 0.56)	− 0.79 (± 0.28)	0.20
CoP pre_comp_EC (mean *z*-score, sd)	0.04 (± 0.61)	− 0.15 (± 0.74)	0.63
CoP pre_comp_tandem (mean *z*-score, sd)	0.83 (± 1.23)	0.32 (± 0.52)	0.39

Overview of characteristics and clinical assessments measured in the first session for 10 responders and 5 non-responders on the contra-lesional cb_tDCS. The assessment range is given in case of ordinal scales. Mean per group are given as well as the standard deviation (sd) or the median and interquartile ranges (iqr) in case of ordinal scales and the frequencies in case of nominal data

*TACI* total anterior circulation infarct, *PACI* partial anterior circulation infarct, *LACI* lacunar anterior circulation infarct, *CIRS* Cumulative Illness Rating Scale, *HADS* Hospital Anxiety and Depression Scale, *BBS* Berg Balance Scale, *TUG* Timed Up and Go, *EmNSA-LE* Erasmus modification of the Nottingham Sensory Assessment Lower Extremity, *FES* Fall Efficacy Scale, *FM-LE* Fugl–Meyer assessment lower extremity, *MI-LE* Motricity Index of the Lower Extremity, *VAS* visual analog scale, *CoP pre_comp* first measured session of the center of pressure composite score for eyes open (*EO*), eyes closed (*EC*), and the tandem stance (*tandem*) position, *z-score* standardized score, *N* number per group

**Table 2:** Overview of the tested models for the effect of stimulation

Association of stimulation on a decrease in CoP comp-score									
Controls (*N* = 10)	Eyes open			Eyes closed			Tandem		
	*β*	95% CI	*p*	*β*	95% CI	*p*	*β*	95% CI	*p*
Intercept	0.24	(-0.00 – 0.49)	0.05	-0.24	(-0.47 to -0.02)	0.04	0.31	(-0.83 – 0.70)	0.12
Pre-stim score	1.17	(0.93 – 1.40)	0.00	-0.01	(− 0.33 – 0.32)	0.96	0.80	(0.57 – 1.03)	0.00
Stimulation	0.02	(-0.09 – 0.12)	0.73	0.08	(− 0.01 – 0.16)	0.07	-0.08	(-0.41 – 0.25)	0.64
Intercept	0.24	(-0.01 – 0.49)	0.06	-0.27	(− 0.47 to -0.06)	0.01	0.33	(-0.27 – 0.93)	0.28
Pre-stim score	1.17	(0.93 – 1.40)	0.00	-0.05	(− 0.47 – 0.30)	0.76	0.79	(0.53 – 1.05)	0.00
Stimulation	0.02	(-0.08 – 0.12)	0.73	0.07	(− 0.01 – 0.16)	0.07	-0.08	(-0.41 – 0.25)	0.64
Order of measurements	0.01	(-0.08 – 0.10)	0.81	0.03	(− 0.06 – 0.11)	0.57	-0.04	(-0.04 – 0.33)	0.84
Patients (*N*= 15)									
Intercept	0.13	(-0.05 – 0.30)	0.15	0.02	(− 0.09 – 0.12)	0.75	0.16	(-0.04 – 0.36)	0.11
Pre-stim score	0.93	(0.74 – 1.13)	0.00	0.76	(0.61 – 0.92)	0.00	0.65	(0.45 – 0.84)	0.00
Ipsi-lesional stimulation	-0.09	(-0.18 to − 0.01)	0.03	-0.01	(− 0.19 – 0.16)	0.89	-0.12	(-0.30 – 0.06)	0.18
Contra-lesional stimulation	-0.07	(-0.19 – 0.06)	0.32	0.02	(− 0.11 – 0.16)	0.73	-0.25	(-0.48 to -0.03)	0.03
Intercept	0.05	(-0.11 – 0.21)	0.54	-0.04	(− 0.20 – 0.12)	0.64	0.25	(0.07 – 0.43)	0.01
Pre-stim score	0.94	(0.79 – 1.09)	0.00	0.75	(0.60 – 0.91)	0.00	0.64	(0.46 – 0.82)	0.00
Ipsi-lesional stimulation	0.00	(-0.09 – 0.90)	0.94	0.09	(−0.08– 0.26)	0.30	-0.08	(-0.55 – 0.40)	0.75
Contra-lesional stimulation	-0.02	(-0.14 – 0.01)	0.71	0.06	(− 0.10 – 0.22)	0.44	-0.26	(-0.43 to -0.09)	0.00
Sham at 3rd session	-0.01	(-0.09 – 0.07)	0.78	-0.06	(− 0.22 – 0.10)	0.44	-0.15	(-0.64 – 0.34)	0.54
Sham at 2th session	0.12	(0.08 – 0.23)	0.04	0.08	(− 0.08 – 0.24)	0.31	-0.11	(-0.29 – 0.06)	0.20
Post hoc analysis of the separate CoP parameters for the tandem position in patients									
	ACoP			varCoP			Range*		
	*β*	95% CI	*p*	*β*	95% CI	*p*	*β*	95% CI	*p*
Intercept	1.31	(0.16 – 2.46)	0.03	1.94	(0.37 – 3.51)	0.02	2.27	(1.40 – 3.66)	0.00
Pre-stim score	0.82	(0.68 – 0.96)	0.00	0.80	(0.63 – 0.96)	0.00	1.26	(1.11 – 1.43)	0.00
Ipsi-lesional stimulation	-0.31	(-1.37 – 0.75)	0.57	-0.30	(− 1.74 – 1.13)	0.68	0.98	(0.89 – 1.06)	0.57
Contra-lesional stimulation	-0.86	(-1.58 to -0.15)	0.02	-1.10	(− 1.93 to -0.26)	0.01	0.94	(0.90 – 0.98)	0.01
Sham at 3rd session	-0.14	(-1.20 – 0.92)	0.80	-0.35	(− 1.79 – 1.10)	0.64	0.97	(0.89 – 1.05)	0.44
Sham at 2nd session	0.01	(-0.72 – 0.75)	0.97	-0.11	(− 0.97 – 0.76)	0.81	0.96	(0.91 – 0.99)	0.04
	VCoP*			varVCoP*					
Intercept	1.38	(1.12 – 1.72)	0.00	1.63	(1.26 – 2.10)	0.00			
Pre-stim score	1.40	(1.30 – 1.50)	0.00	1.32	(1.21 – 1.44)	0.00			
Ipsi-lesional stimulation	0.98	(0.87 – 1.1)	0.68	0.98	(0.85 – 1.12)	0.73			
Contra-lesional stimulation	0.97	(0.94 – 0.99)	0.02	0.97	(0.93 – 1.01)	0.11			
Sham at 3rd session	1.00	(0.89 – 1.12)	0.96	0.98	(0.86 – 1.12)	0.78			
Sham at 2nd session	0.98	(0.95 – 1.00)	0.07	0.96	(0.92 – 1.01)	0.08			

Overview of the generalized estimating equation models that were tested for the effect of stimulation on post-measurement center of pressure (CoP) parameters in healthy controls and patients. All models were corrected for the individual pre-measurement CoP scores. If the *β* values in the model changed more than 10% by correcting for the randomized order of the stimulation to the model, this was considered an improvement of the model. Sham stimulation condition or sham stimulation condition at the first session was used as contrast. Parameters marked with an asterisk (*) were natural logarithmic transformed, the exponential functions of *β* and the CI are given in the table and should be interpreted as ratios

*ACoP* the mean amplitude of the CoP, variability of the mean amplitude of the CoP (*varCoP*), *VCoP* the velocity of the CoP, variability of the velocity of the CoP (*varVCoP*), *β* unstandardized beta value, *CI* confidence interval

## Discussion

To our knowledge, this is the first study reporting the short-term effect of cb_tDCS on standing balance performance in patients with chronic stroke. The effect of cb_tDCS was tested during three static positions, namely, eyes open, eyes closed, and in a tandem position. No effects of cb_tDCS on standing balance performance were found in the first two positions for patients nor in control subjects. In the tandem stance position, a significant decrease in four separate CoP parameters and in the CoP comp-score was found, suggesting an improvement in standing balance performance in the stroke patients after contra-lesional anodal stimulation. In healthy controls, no effect of cb_tDCS was found in the tandem stance position.

tDCS is believed to facilitate motor learning while being simultaneously applied with a motor task (40). It could therefore be expected that the most difficult task, the (semi-)tandem position in which there is most to gain, would show the largest improvement in standing balance performance after training with cb_tDCS. It is likely that the tandem stance position and the tracking task were not difficult enough for the healthy controls. Similar to the study by Steiner et al. (24), who used a moving platform to train balance performance in 30 healthy young adults and found no effects of cb_tDCS, healthy subjects in our study were also likely performing on a (sub)optimal level with a mean performance of 98.5% after the first session and had very little room for improvement. Future studies with an interest in the feasibility of cb_tDCS in healthy subjects should use a more challenging postural task or a dual task paradigm.

Poortvliet et al. (22) found a significant smaller CoP path length and standard deviation during and after disturbed proprioceptive input with Achilles tendon vibration in quiet stance, in the group receiving cb_tDCS as compared to sham stimulation. This is an interesting model in the understanding of cb_tDCS on balance performance since dysfunction of proprioception can occur after stroke (41) and can hamper balance performance. It would be interesting to study whether proprioceptive function is an independent covariate for improvement of balance performance after cb_tDCS in patients after stroke. Unfortunately, the sample size in the current study was too small to perform such a sub-analysis. The reported changes in standing balance performance in the tandem stance position with contra-lesional stimulation in patients with a stroke are in line with the theoretical framework that both LTP- and LTD-like processes play a role in spike timing-dependent forms of neuroplasticity in the cerebellum and non-invasive stimulation may enhance these processes (7). Theoretically, stimulation on either one of the cerebellar hemispheres could enhance adaptive motor learning and thereby improve motor coordination. Targeting the contra-lesional cerebellar hemisphere could directly strengthen the M1–cerebellar connection, to enhance the function of the cerebellum connected to the affected cortical hemisphere (15, 23). On the other hand, neural activity of the ipsilateral cortex during movements has been associated with poor functional outcome after stroke (42). This phenomenon has been attributed to a decreased inter-hemispheric inhibition (IHI) from the lesioned motor cortex on the non-affected hemisphere, leading to an increased IHI on the affected hemisphere, negatively affecting functional outcome (43). Inhibition of the ipsi-lateral motor cortex with cathodal stimulation is thought to suppress this overactivity (44). Anodal stimulation of the ipsi-lesional cerebellar hemisphere could have potentially improved the disturbed IHI in an indirect manner via cerebellar brain inhibition and at the same time enhance adaptive learning. The results of this study did not show evidence that targeting IHI via the cerebellum can lead to improvement in terms of standing balance performance. From these results, it cannot yet be concluded if the protocol failed to induce a normalization in IHI or if it should be considered a compensatory mechanism reflecting the severity of the brain damage, in which normalization of this phenomena does not have any added value for clinical outcome (45). Future research should aim to underpin the neurobiological mechanisms by which cb_tDCS enforces its effects.

### Baseline Differences Between Patients and Healthy Controls

The increase in postural sway found in stroke patients compared to aged-matched controls and the enlarged sway with the more difficult positions are in line with previously found results in a comparable population (46). However, no significant differences in CoP parameters were found between stroke patients and controls for the tandem stance position. This result may be explained by the individualized feet positioning during the tandem stance per subject. None of the stroke patients could hold the full tandem stance position with either leg behind, while all but one healthy subject could hold this position with the non-preferred leg behind. This again indicates that the task may not have been difficult enough for the healthy controls. The main purpose of this proof-of-concept study was to investigate the potential of cb_tDCS to elicit qualitative changes in standing balance performance in patients with a stroke. Since the differences between patients are much larger than the effect that can be expected from a single training session, a within-subject design was needed with a challenging task, tailored to the specific capacity of each patient.

### Outcome Parameters

A decrease in CoP parameters is generally assumed to reflect an improvement in postural stability in patients with a stroke (47). There is a strong need for more sensitive and reliable measures to be able to quantify subtle changes in standing balance performance and disentangle postural control mechanisms (48). Despite several promising methods to quantify postural control, a golden standard is still lacking (49, 50). We used the CoP comp-score as a sensitive comprehensive outcome parameter, combining information from five parameters of standing balance performance (39). A sensitive outcome parameter, able to detect subtle qualitative changes, is also needed to establish an optimal dose in terms of sessions and intensity. To relate the currently found short-term effects of anodal cb_tDCS on standing balance performance to a clinical meaningful and long-term improvement, the effect of multiple training sessions should be measured using both qualitative parameters of standing balance performance and clinical outcome measures.

### Responders and Non-Responders

A general point of concern in tDCS research is the different responsiveness of subjects to the stimulation, i.e., why do some subjects respond to the stimulation while others do not? Several reviews on both healthy subjects as well as patients with stroke have highlighted factors such as age, anxiety, time since stroke, lesion type, lesion location, and motor function as contributors to interindividual variability in response to tDCS (51–55). Within this study, ten patients showed changes toward normal CoP values in response to contra-lesional cb_tDCS compared to sham. On a group level, this contrast was large enough for a significant association; the non-responders should, however, not be ignored. These interindividual differences play a role in many tDCS studies, which lead to ambiguity in the interpretation of results and the conclusion of some authors that tDCS does not have any added value or vice versa (56). Within the small sample of this study, no differences could be detected between responders and non-responders on several subject characteristics. Next to clinical characteristics, the initial state of neuronal populations could play a role in terms of responsiveness to the stimulation (54, 57). Neuroimaging techniques could provide valuable insights into these neuronal state dependencies (58).

### Strength and Limitations

This proof-of-concept study is the first aimed at the short-term effect on standing balance performance of a single training sessions combined with anodal cb_tDCS in stroke patients. The current setup was able to detect subtle qualitative changes in standing balance performance by using high-resolution kinetic parameters. The study population was however small and heterogeneity in terms of lesion type, location, and motor function could have influenced the interindividual variability in response to the stimulation. Within the sample, two patients were included with a lesion primarily located in the brainstem; both of these patients were classified as responders to the contra-lesional cb_tDCS. The current sample is, however, too small to perform a sub-group analysis or to generalize these findings to a wider population. The healthy controls in this study only received stimulation ipsi-lateral to their dominant leg. It is possible that an improvement in the healthy subjects could have been found after contralateral stimulation. The more evident explanation for the lack of improvement in healthy controls is, however, that the task was too easy and a more challenging task should have been chosen for this group.

### Future Research

The current study shows the potential of anodal cb_tDCS for improving standing balance performance in a chronic stroke population, though interindividual differences should be further studied. Analysis of ongoing cortical processes during standing balance tasks and the influence of cb_tDCS on these processes may give more insights in unknown underlying mechanisms. Moreover, these unknown mechanisms leading to interindividual differences could be very different in the more acute phase after stroke. Behavioral restitution of function, mainly taking place in the first 8–12 weeks post-stroke (59), go alongside changes in growth factors, creating a critical time window for recovery (60–62). tDCS might be able to optimize the learning potential in this time window, leading to a completely different paradigm of the mechanisms of action of tDCS in the sub-acute phase than in a population of patients in a chronic phase after stroke (63, 64). Considering that balance recovery is especially important in the early phase after stroke, tDCS interventions should also be tested in this time window of enhanced neuroplasticity. In addition, possible contributors to interindividual differences, such as a wide variety of clinical characteristics as well as neuronal state, should be recorded.

## Conclusion

The improvement in standing balance performance after anodal contra-lesional cerebellar tDCS shows promise for the application in stroke rehabilitation. Future studies should investigate interindividual differences to elucidate the working mechanisms of tDCS. Qualitative outcome parameters that can capture the subtle effects of tDCS can be used to explore the optimal dose and should be related to clinically meaningful improvements. High-quality randomized controlled trials in the early phase after stroke are needed to establish the role of cb_tDCS in the critical time window of recovery after stroke and its potential to enhance clinical outcome in rehabilitation practice.
